# Polymeric Hydrogels Loaded with ZnO Nanoparticles as Promising Tools for Tacking Viral Skin Disorders

**DOI:** 10.3390/v18010076

**Published:** 2026-01-05

**Authors:** Ana Karen Jaimes, Victoria Ayala-Peña, Agustín Buzzi, Vera Álvarez, Verónica Lassalle

**Affiliations:** 1 INQUISUR, Departamento de Química, Universidad Nacional del Sur, Av. Alem 1253, Bahía Blanca B8000, Argentina; anak.jaimes.97@gmail.com; 2 Departamento de Biología, Bioquímica y Farmacia, Universidad Nacional del Sur (UNS)-CONICET, San Juan 670, Buenos Aires B1001, Argentina; vayala@criba.edu.ar (V.A.-P.); agusbuzzi@gmail.com (A.B.); 3 Instituto de Investigaciones en Ciencia y Tecnología de Materiales (INTEMA), Universidad Nacional de Mar del Plata (UNMdP)-CONICET, Avenida Colón 10850, Mar del Plata B7600, Argentina; veraalejandraalvarez@gmail.com

**Keywords:** zinc oxide nanoparticles, biopolymeric hydrogels, antiviral activity

## Abstract

Zinc oxide nanoparticles (ZnO NPs) have attracted growing interest in several fields, including topical biomedical applications, due to their stability, biocompatibility and therapeutic potential. In this study, chitosan (Ch), gelatin (G) and arabic gum (AG) were combined to formulate hydrogels loaded with different ZnO NP concentrations. The main aim is to assess the synergy between the properties of biopolymers and ZnO moieties in terms of antiviral activity. ZnO NPs were synthesized via co-precipitation. Hydrogels were prepared using the freeze–thaw method, and the loading of 2.5, 5 and 7.5% *w*/*w* of ZnO NPs with respect to Ch was promoted by ultrasonication. The structural, morphological, surface and thermal properties of hydrogels loaded with ZnO NPs (HZ 2.5, HZ 5 and HZ 7.5) and the control matrix (H) were characterized using FTIR spectroscopy, confirming the successful incorporation and interaction of ZnO NPs with the polymeric network. Low ZnO NP concentrations enhanced the swelling degree of the hydrogels (from 1044% to 1253%), improving their thermal stability and solubility (96 h vs. 48 h HZ 7.5 and 14 h in the case of H). This behavior could be ascribed to the aggregation of ZnO NPs with increasing amounts, which was verified through FESEM. Virucidal activity was tested against herpes simplex virus type 1 (HSV-1) and bovine coronavirus (BCoV), demonstrating a substantial enhancement when the ZnO NPs are added independently of the concentration. An almost 100% viral inhibition was recorded when the HZs were analyzed, whereas the H matrix showed an inhibition of about 40% against the same virus. Antioxidant activity was evaluated via the DPPH free radical inhibition method, revealing an improvement with the loading of NPs.

## 1. Introduction

Viral skin disorders have a significant impact on the global population, both in terms of public health and socioeconomic consequences. In 2021, cutaneous viral infections exhibited a global incidence of 84.7 million cases and a prevalence of 136.8 million, predominantly affecting children and adolescents, exhibiting higher rates in more crowded regions [1]. The severity of these conditions can range from mild, self-limiting discomfort to complex chronic diseases. As secondary effects, these dermatological disorders may lead to anxiety, depression and a reduced quality of life due to the spreadable nature of the infections, causing, in addition to discomfort and pain, visible skin lesions and the related social stigma [2].

Some of the most common viral skin conditions are caused by herpes simplex viruses (HSV-1 and HSV-2) acutely or by its reactivations, herpes zoster, resulting from the reactivation of the varicella-zoster virus (VZV), human papillomavirus (HPV) responsible for warts, parvovirus B19, which causes erythema infectious, and poxviruses of the Molluscipox genus, which also cause molluscum contagiosum, among others [3,4,5,6,7]. Even when these diseases affect a vast group of the population, immunocompromised patients or persons with chronic skin disorders are included in the highest risk group for developing cutaneous infections, including secondary bacterial and viral infections. This situation has driven the urgent need to develop innovative, more effective, safe and low-cost therapeutic alternatives. In this context, polymeric hydrogels appear as a promising approach for improving the management of viral skin disorders.

Gelatin (G), a naturally derived polymer, has been reported as a suitable option for the fabrication of hydrogels intended for biomedical applications due to its biocompatibility, biodegradability and non-toxicity. Its tunable porosity allows for the encapsulation of drugs within the gel structure and their controlled release [8]. Despite these advantages, G possesses low mechanical strength, undergoing a rapid degradation under physiological conditions [9,10]. These limitations can be solved by exploiting the variety of functional groups in G’s structure that enable facile crosslinking with other polymers and crosslinking agents. Chitosan (Ch), a cationic polysaccharide derived from chitin, exhibits antimicrobial, antiviral, antioxidant and antitumor properties [11,12,13,14,15]. However, its biomedical use is hindered by its limited solubility in aqueous media and poor mechanical properties. Ch provides bioactivity and also enhances the hydrogel’s stability, resistance to degradation and mechanical strength, while improving its performance in controlled drug release systems when incorporated into G-based hydrogel formulations [16,17]. Additionally, this combination provides several advantages, including thermoreversible gelation, transparency, non-toxicity, non-antigenicity, non-immunogenicity, biocompatibility, biodegradability and biosafety [18,19,20,21]. The formation of chemically crosslinked hydrogels requires the presence of a crosslinking agent. Arabic gum (AG), an anionic polysaccharide with carboxyl groups, can engage in electrostatic interactions with G under acidic conditions, forming coacervate complexes that enhance structural cohesion and water retention. Ch, as a cationic polysaccharide with amino groups, can form both ionic and hydrogen bonds with G and AG, thereby reinforcing the hydrogel network and improving its mechanical properties and degradation resistance [22]. This combination of polymers is expected to also increase biofunctionality [18,23], supporting its application in dermatological treatments ranging from chronic skin conditions to skin regeneration after invasive procedures.

The advance of nanotechnology has revolutionized the field of biomaterials. In particular, the uses of ZnO NPs have been widely documented, making them highly attractive for biomedical applications. Studies reported the lack of cytotoxicity at concentrations up to 20 mg/L [24,25,26]. The current evidence indicates that these particles do not pose a health hazard when topically applied, as they do not penetrate intact or compromised human skin. The United States Food and Drug Administration (FDA) has classified ZnO as a substance generally recognized as safe (GRAS), supporting its use in dermatological and pharmaceutical products [27,28,29]. These nanomaterials have proven to be a promising option due to their antimicrobial, antiviral, antifungal, antioxidant, anti-inflammatory and anticancer properties [29]. Moreover, zinc plays a crucial role in modulating the immune response and helping regulate viral replication [30,31].

It has been reported that the incorporation of ZnO NPs embedded into polymeric matrixes influences their water absorption capacity, morphology, stability, biodegradability and bioactivity, while demonstrating that these modifications are dose-dependent [32,33,34,35]. Therefore, determining the optimal nanoparticle concentration is critical for controlling the properties of biopolymeric hydrogels intended for biomedical applications. Yadollahi et al. [36] developed Ch-based hydrogels incorporating various concentrations of in situ synthesized ZnO NPs, resulting in enhanced physicochemical properties of the matrix, such as modified swelling behavior and optimized controlled drug release. These improvements position the hydrogels as promising candidates for drug delivery system applications. Yu et al. [37] synthesized ZnO NPs in situ within G hydrogels, leading to improved thermal stability and mechanical properties of the material. These enhancements were attributed to non-covalent interactions between ZnO NPs and the polypeptide chains of G. Furthermore, the incorporation of NPs provided antibacterial activity. Emir Coban et al. [38] investigated Ch-based biofilms containing ZnO NPs, observing that their incorporation reduced the water retention capacity of the films while enhancing the thermal properties of the formulation.

Recent advances in hydrogel-based biomaterials have emphasized their role as multifunctional platforms capable of integrating structural support with biological activity in complex therapeutic scenarios. High-impact studies have demonstrated that tailoring hydrogel composition, network architecture and functional additives is essential to modulate bioactivity, stability and interactions with biological environments, particularly for skin-related applications [39,40]. These works highlight the growing interest in designing advanced hydrogel systems that combine physicochemical tunability with therapeutic functionality.

Despite these advances, comprehensive studies systematically correlating nanoparticle loading with hydrogel microstructure, physicochemical stability and biofunctional performance under skin-mimicking conditions are still missing. In particular, the concentration-dependent effects of ZnO NPs on swelling behavior, network integrity, surface properties, antioxidant activity and antiviral potential have not been simultaneously addressed within a single biopolymeric platform.

In this context, this study aims to design and characterize multifunctional hydrogels composed of G, Ch and AG incorporating ZnO nanoparticles, focusing on how nanoparticle loading affects the physicochemical and functional properties of the matrix. Viral skin infections trigger oxidative stress and inflammation, intensifying tissue damage; hence, hydrogels with antioxidant and antimicrobial potential are promising for topical applications.

The goal of this work is to develop a multifunctional and versatile hydrogel with the potential to treat viral skin disorders, taking advantage of the properties of G, Ch and AG biopolymers combined with ZnO NPs. Viral cutaneous infections are known to induce oxidative stress and a robust inflammatory response, exacerbating tissue damage and hindering regeneration. In this context, the use of a hydrogel possessing dual antiviral and antioxidant activity could derive a more efficient and innovative therapeutic strategy.

Even when ZnO NPs have been accepted by the regulatory entities (FDA, EMA), recommended doses exist for its application in cosmetics, medicine and food. So, exploring the range of ZnO NP doses able to induce improvements in H properties is a key research point. This contribution presents the synthesis and characterization of G, Ch and AG hydrogels, focusing on the influence of ZnO NP loadings on the properties of interest, such as the thermal ones, swelling degree, solubility, gel fraction and hydrophobicity character, among others. The antiviral and antioxidant activities of the formulations have also been evaluated in this work, aiming to provide preliminary evidence of their bioactive potential.

## 2. Materials and Methods

### 2.1. Materials

The chitosan (Ch) used in this study was obtained from Farmacia Homeopática Pereda, Mar del Plata, Buenos Aires, Argentina. Gelatin (G) was supplied by Fluka Analytical (Buenos Aires, Argentina), and arabic gum (AG) was supplied by Biopack, Buenos Aires, Argentina. The acetic acid was provided by Cicarelli. ZnSO_4_.H_2_O (Cicarelli, Buenos Aires, Argentina) and NaOH (Anedra, Buenos Aires, Argentina) were used as precursors during the synthesis of ZnO NPs. DPPH (2,2-Diphenyl-1-picrylhydrazyl) was purchased from Sigma Aldrich (Buenos Aires, Argentina).

### 2.2. Synthesis of ZnO NPs

**Figure 1:**
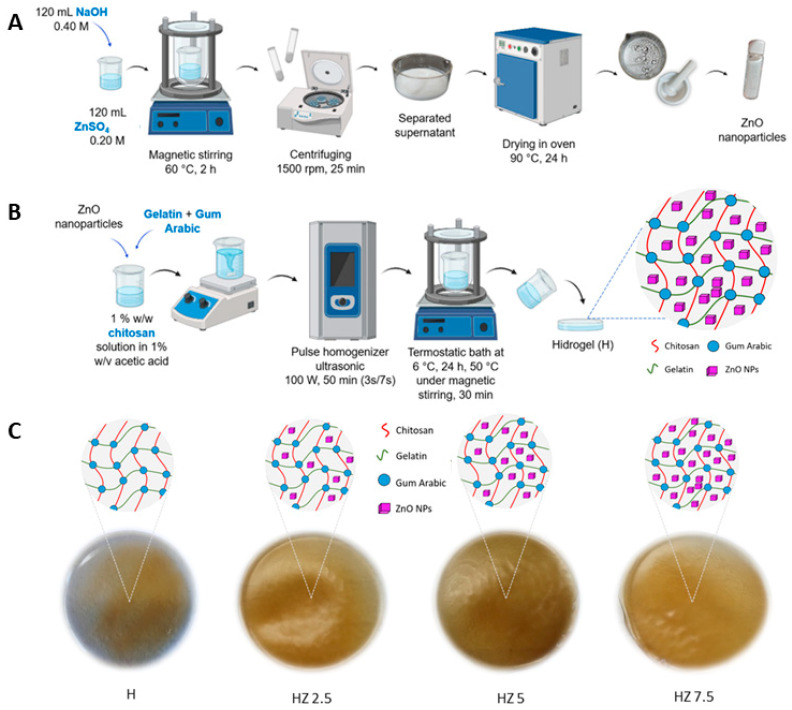
(**A**) Synthesis of ZnO NPs. (**B**) Hydrogel synthesis and encapsulation of ZnO NPs, which become entrapped within the polymeric network formed by Ch, G and AG. (**C**) Schematic of the matrix (H) and hydrogels with different concentrations of ZnO NPs (HZ 2.5, HZ 5 and HZ 7.5).

ZnO NPs were synthesized via the coprecipitation method, following the procedure described by Perez Adassus et al. [41], which is illustrated in Figure 1A. Specifically, 120 mL of 0.20 M ZnSO_4_ solution was heated to 60 °C. Then, 120 mL of 0.40 M NaOH solution was added dropwise under continuous stirring at a flow rate of 1 mL/min. The mixture was kept under magnetic stirring at a constant temperature for 2 h. The resulting white dispersion was centrifuged at 1500 rpm for 30 min. After centrifugation, the supernatant was discarded. No washing steps were performed. The solid was transferred to a pre-weighed crystallizer and dried in an oven at 90 °C for 24 h. The dried material was then ground using a mortar to obtain a fine powder. The synthesized ZnO NPs were thoroughly characterized in Perez Adassus et al. [41], and most representative data is included in Figure S1 of the Supplementary Materials.

### 2.3. Preparation of Hydrogels

Hydrogels (H) were synthesized using the freezing–thawing method previously used in our own published articles [42]. ZnO NPs were encapsulated within the hydrogels through ultrasonication, according to the procedure schematized in Figure 1B. To prepare the hydrogel, a precursor solution was prepared by dissolving 0.25 g of Ch in 25 mL of a 1% (*w*/*v*) acetic acid solution. The Ch solution thus obtained also had a final concentration of 1% (*w*/*v*), and the mixture was stirred magnetically for several minutes. Subsequently, 0.5 g of G and 0.075 g of AG were added, along with ZnO NPs. The added NP concentration ranged between 2.5 and 7.5% *w*/*w* with respect to the Ch mass. The mixture was subjected to pulsed ultrasound irradiation (3 s on 7 s off) at a power of 100 W for 50 min by using a pulsed ultrasonic homogenizer (DP0150-6, Benchmark Scientific, Sayreville, NJ, USA) equipped with a 6 mm diameter titanium probe (150 W, 75% efficiency). The temperature was controlled using a static water-cooling bath, maintaining room temperature throughout the process. The resulting mixture was placed in a thermostatic bath at 6 °C for 24 h. The bath temperature was then increased up to 50 °C, and the mixture was stirred for 30 min. After turning off the agitation and the bath, the mixture was poured into a mold and left at room temperature until complete solvent evaporation. This process yielded the unloaded hydrogel matrix (H) and was repeated, adding the mentioned ZnO NP concentrations. The summarized information is listed in Table 1.

### 2.4. Characterization of the Hydrogels

#### 2.4.1. FT-IR Spectroscopy

A Thermo Scientific Nicolet iS50 FTIR spectrophotometer was used in transmission mode, operating at a frequency range between 400 and 4000 cm^−1^ to determine qualitatively the composition of hydrogels. Compact pellets of H, ZnO NPs and H loaded with different concentrations of ZnO NPs were prepared by mixing the samples with KBr.

#### 2.4.2. Gel Fraction

To determine the gel fraction, the samples were dried in an oven at 37 °C for 48 h and then weighed (*Wi*). Subsequently, they were immersed in distilled water for 3 days inside a cellulose dialysis membrane to prevent material loss during weighing. The samples were then re-dried at 37 °C for another 48 h and weighed again (*Wf*) [42]. The gel fraction was calculated using the following equation:

(1) $$GF\%=\frac{Wf}{Wi}\times 100\%$$

Gel fraction (%).

#### 2.4.3. Determination of Solubility

To determine the time required for hydrogel’s complete dissolution, 40 mg of each gel was placed in a cellulose dialysis membrane immersed in 30 mL of acetate buffer (pH 5.22) at 32 °C, simulating the cutaneous environment.

#### 2.4.4. Contact Angle Measurements

The hydrophobicity and wettability of the hydrogel surfaces were evaluated by measuring the advancing water contact angle at room temperature using an OCA 15 goniometer. A droplet of bidistilled water was deposited on the sample surface, and its contour at equilibrium was analyzed with the instrument software. Each reported value represents the average of three measurements taken at different sample areas while monitoring the droplet behavior in real time.

#### 2.4.5. Swelling Degree (SD)

Swelling behavior was evaluated gravimetrically in acetate buffer (pH 5.22) at room temperature to simulate the dermal environment. Dried hydrogel samples (*Wi*) were immersed in the buffer and periodically weighed (*Wf*) after blotting until equilibrium was reached [42]. The swelling degree (*SD* %) was then calculated using the corresponding equation:

(2) $$SD\%=\frac{\left(Wf-Wi\right)}{Wi}\times 100$$

Swelling degree (%).

#### 2.4.6. Field Emission Scanning Electron Microscopy (FESEM) and Energy-Dispersive X-Ray Spectroscopy (EDS)

The microstructure, surface characteristics and morphology of the hydrogels were examined using FESEM (ZEISS Crossbeam 350, Jena, Germany) after chromium coating. Surface and cross-sectional images were captured at magnifications from 500× to 30,000×. The presence of ZnO NPs was confirmed using EDS.

#### 2.4.7. Thermal Properties: Differential Scanning Calorimetry (DSC) and Thermogravimetric Analysis (TGA)

The thermal stability of the hydrogels was evaluated through DSC and TGA. DSC thermograms were recorded from −20 to 220 °C at a heating rate of 10 °C/min using a DSC Q2000 (TA Instruments, New Castle, DE, USA). TGA analyses were carried out from 0 °C to 780 °C under a nitrogen atmosphere (40 mL/min) with the same heating rate, using approximately 10 mg of sample.

#### 2.4.8. Flame Atomic Absorption Spectroscopy (FAAS)

The determination of Zn to estimate the amount of ZnO NPs loaded at HZ 2.5, HZ 5 and HZ 7.5 was performed using a GBC Avanta 932 atomic absorption spectrometer (Offenburg, Germany). Acetylene gas and a zinc lamp were used to determine the metal concentration. For this purpose, 40 mg of the samples was completely disaggregated in 30 mL of hydrochloric acid (HCl, 37% *w*/*w*) and measured.

### 2.5. Biological Studies

#### 2.5.1. Cytotoxicity Analysis

The cytotoxicity of the hydrogel HZ 7.5 was evaluated using the neutral red uptake assay (NRU), following the ISO 10993–5: 2009 guideline as before [16,43]. The cytotoxicity was indirectly evaluated through the incubation of the cells with the extracted medium of the hydrogels. Briefly, after adding 2 × 2 mm^2^ fragments to 5 mL of culture media, the mixture was cultured for 24 h at 37 °C. Concentrations of 25 to 100% of the discharged media were diluted and added to the cells. In parallel, 96-well plates were seeded with 2 × 10^5^ cells/well in triplicate. After that, the cells were exposed to progressively higher concentrations of the substance extracts for a full day. The cells were incubated in the culture medium as a control, and 1% hydrogen peroxide was added to the culture medium as a positive control of cell death. Following treatment, the cells were cultured with neutral red media (NR medium) (1 mL stock solution 3% NR in 79 mL of DMEM 1.5% SFB) after being cleaned with phosphate buffer of pH 7.4 at 37 °C. Following a three-hour incubation period with the NR media, the cells were rinsed with phosphate buffer, and the dye that the cells had ingested was extracted using a 1% acetic acid, 50% distilled water and 49% ethanol 96° solution. Lastly, a Biotek Synergy HT plate reader (BioTek Instruments, Inc., Winooski, VT, USA) was used to quantify the absorbance at 540 nm.

#### 2.5.2. Cell Culture

Vero cells (ATCC^®^ CCL-81) and HRT-18 (ATCC^®^ CCL-244) were grown in Dulbecco Eagle’s medium (D-MEM, Gibco™ Thermo Fisher Scientific, Waltham, MA, USA) supplemented with 5% fetal bovine serum (Gibco™ Thermo Fisher Scientific, Waltham, MA, USA), 0.5% gentamicin (Sigma-Aldrich, St. Louis, MO, USA) and 1.5 g/L of sodium bicarbonate. Cell cultures were incubated at 37 °C and 5% CO_2_. Cells were planted at a density that resulted in 90% confluency following a 24 h incubation period.

#### 2.5.3. Viruses Stock

The experiments were performed using human herpes simplex type 1 (HSV-1) strain Kos and bovine coronavirus (BCoV) strain Mebus. BCoV and HSV-1 viral stock preparation were performed in human rectal adenocarcinoma cells (ATCC^®^ HRT-18) and in Vero cells (ATCC^®^ CCL-81), respectively.

#### 2.5.4. Determination of Antiviral Properties

The virucidal activity of different H with and without ZnO NPs was evaluated using BCoV and HSV-1. A total of 20 µL of solution containing 10^3^ PFUs (plaque-forming units) or mock was inoculated into each 1 cm^2^ of hydrogel and incubated for 15 min at 25 °C in a droplet infection simulation. Next, the remaining viral activity was measured using a plaque assay in Vero cells, as reported before [16,44]. After incubation, PFUs were counted for each treatment. The following formula was applied in order to calculate the percentage of PFU reduction:

% reduction = 100 − [(average viral plaques in the control condition − average viral plaques in the treated condition)/(average viral plaques in the control condition)] ×100.

### 2.6. Determination of Antioxidant Activity

The antioxidant activity of the hydrogels was evaluated using the DPPH free radical scavenging assay according to M. Blois [45]. DPPH is a highly stable free radical due to the delocalization of its unpaired electron, which imparts a deep violet color to its ethanolic solution, characterized by an absorption band centered approximately at 517 nm. When the solution is exposed to a substance capable of donating a hydrogen atom, DPPH is reduced and loses its characteristic violet color [46]. This decolorization can be measured using UV spectroscopy, and the DPPH radical scavenging percentage can then be calculated using the following equation:

(3) $$\%DPPH\;scavenging=\frac{\left(Absi-Absf\right)}{Absi}\times 100\%$$

*DPPH Scavenging* (%).

Where *Absi* is the absorbance of the ethanolic DPPH solution at 517 nm, and *Absf* is the absorbance at 517 nm after contact with the hydrogels.

A stock solution of DPPH radicals (10^−3^ M) was prepared in ethanol prior to the analysis. This solution was protected from light using aluminum foil. The absorbance values were adjusted to 1.00 ± 0.200 [47]. In total, 5 mg of H, HZ 2.5, HZ 5 and HZ 7.5 was mixed with 3 mL of the DPPH solution, shaken and incubated in the dark for 30 min. Afterward, the samples were filtered, and the absorbance was measured using UV/vis spectroscopy at λ = 517 nm to determine the DPPH scavenging percentage. A reference sample containing 3 mL of the DPPH solution and absolute ethanol as a blank was also prepared. Ascorbic acid was used as a qualitative positive control in the assay to confirm the reliability of the DPPH.

### 2.7. Statistical Analysis

Statistical analysis was performed using GraphPad Prism software (version 5.01). All experiments were carried out at least in triplicate, and the results are presented as mean ± standard deviation (SD). Statistical significance among groups was evaluated using analysis of variance (ANOVA). Differences were considered statistically significant at *p* < 0.05.

## 3. Results

### 3.1. Characterization of H and ZnO NPs Loaded H

#### 3.1.1. FT-IR Spectroscopy

**Figure 2:**
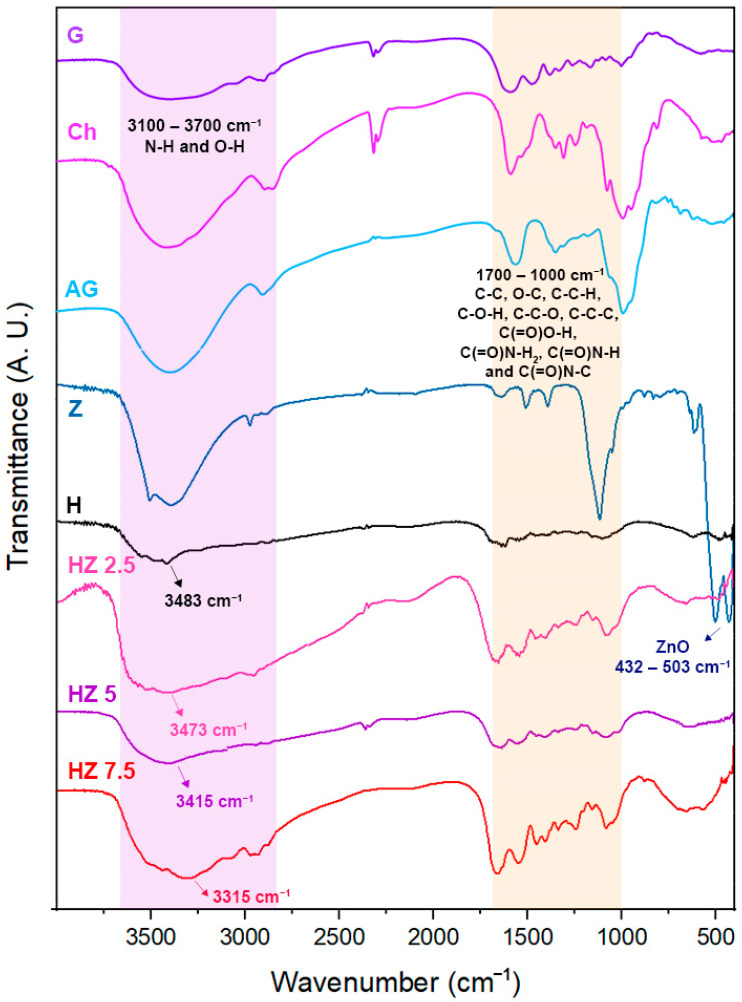
A. FTIR spectra corresponding to G, Ch, AG, Z, H and HZ 2.5, HZ 5 and HZ 7.5.

The incorporation of a crosslinking agent was required in order to reach materials stable enough under the application conditions. In order to investigate the possibility of structural changes in G molecules in hydrogels due to interactions with AG and Ch, and to corroborate the presence of ZnO NPs, FTIR spectroscopy was performed. In Figure 2, the spectra of raw biopolymers and ZnO NPs are compared with those corresponding to H and ZnO NP-loaded H.

#### 3.1.2. Gel Fraction

The degree of crosslinking between polymer chains in a hydrogel is a critical parameter directly influencing some properties of interest, such as mechanical and functional stability. The insoluble fraction, determined through the gel fraction assay, corresponds to the percentage of polymer that is crosslinked, forming a stable three-dimensional network that does not dissolve in a given solvent, which in this case is water. Conversely, the soluble fraction represents the free or partially crosslinked polymer chains that are released to the media during the assay. The gel fraction data are listed in Table 2.

#### 3.1.3. Determination of Solubility

The dissolution data of hydrogels containing different concentrations of ZnO NPs are included in Table 2.

#### 3.1.4. Contact Angle Measurements

**Figure 3:**
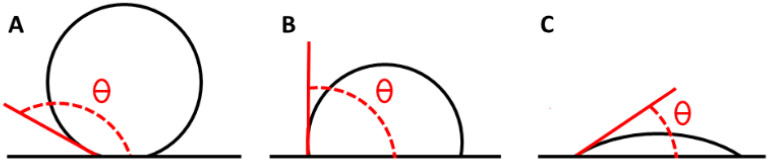
Contact angle of a superhydrophobic material (**A**), in a state of hydrophobic–hydrophilic equilibrium (**B**) and of a hydrophilic material (**C**).

The surface wettability and hydrophobicity can be characterized by measuring the advancing water contact angle, which depends on the balance between cohesive and adhesive forces between water molecules and the H surfaces. Wettability is determined through the surface energy, which depends on physical parameters such as roughness and chemical parameters such as the surface composition. Surfaces exhibiting contact angles above 150° (Figure 3A) are typically classified as superhydrophobic. A value of contact angle of 90° indicates a balance between hydrophilic and hydrophobic characteristics, suggesting an intermediate affinity of the material for the wetting liquid. In this case, the material does not exhibit a strong attraction to, nor a significant repulsion from, the liquid (Figure 3B), while a contact angle below 90° is considered a hydrophilic surface (Figure 3C) [48].

**Figure 4:**
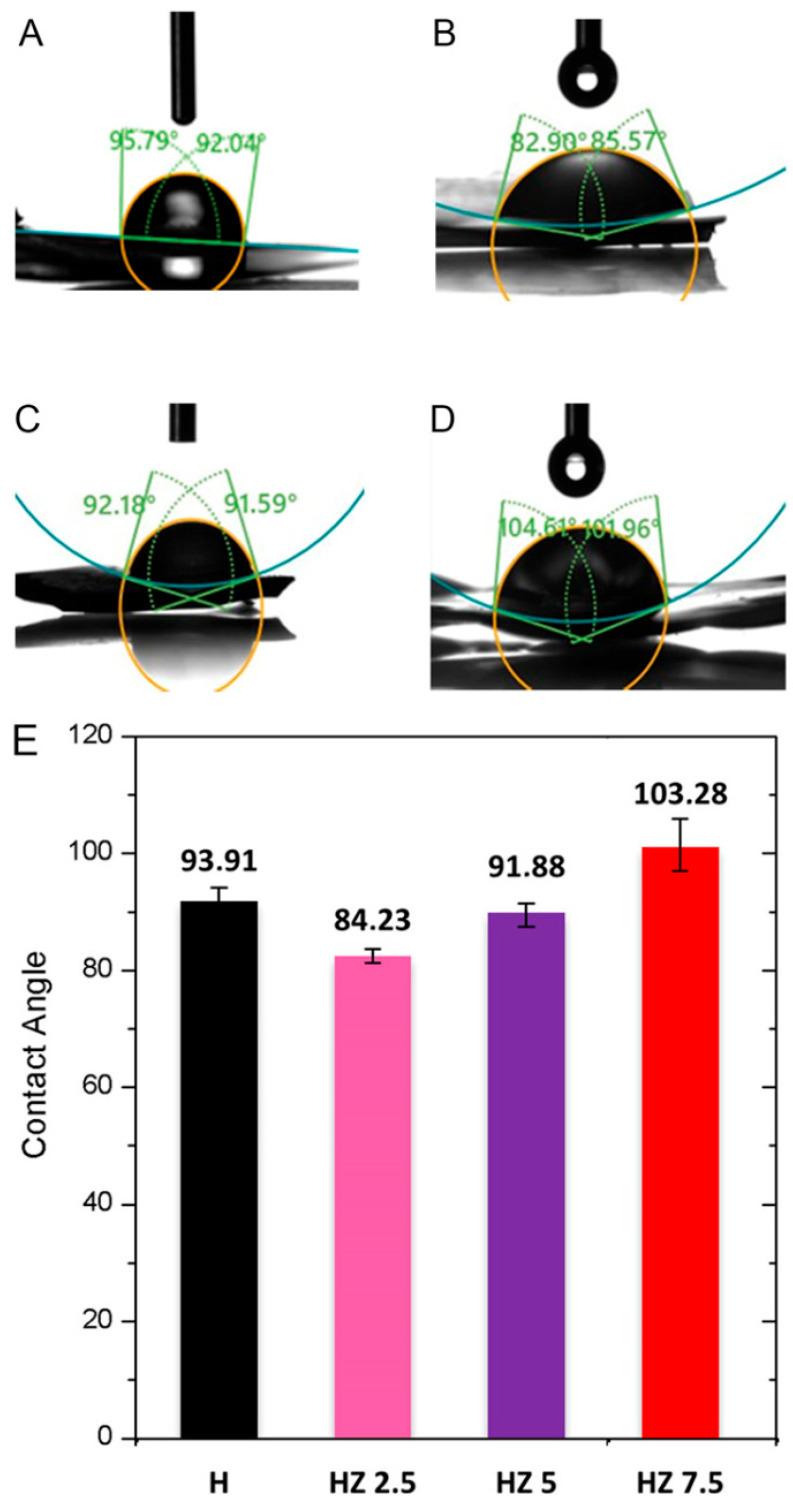
Advancing water contact angle of H (**A**), HZ 2.5 (**B**), HZ 5 (**C**) and HZ 7.5 (**D**). Comparison of the average advancing water contact angle for the different samples (**E**).

Figure 4 displays images of the water contact angles for H (A), HZ 2.5 (B), HZ 5 (C) and HZ 7.5 (D). Figure 4E presents the average advancing water contact angle for each sample.

#### 3.1.5. Swelling Degree (SD)

**Figure 5:**
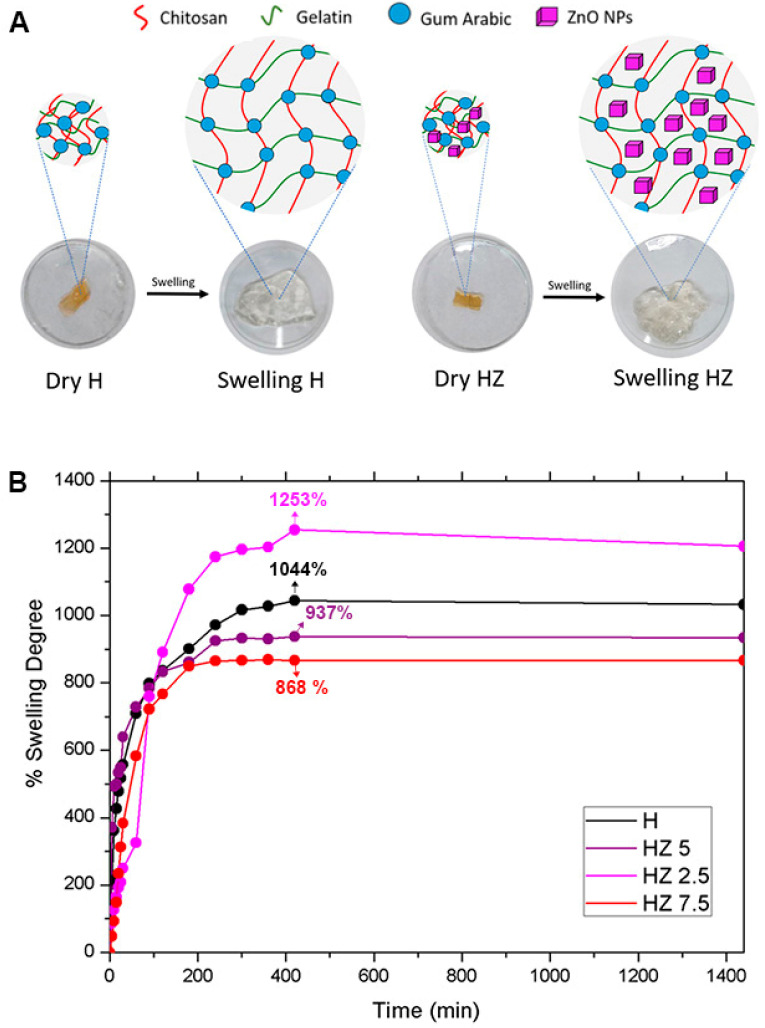
(**A**) Dry state and swollen state of H after aqueous solvent uptake. (**B**) Swelling degree of H, HZ 2.5, HZ 5 and HZ 7.5.

To investigate the water absorption capacity of the hydrogels and whether this property is affected by the incorporation of varying concentrations of ZnO NPs, the swelling degree was assessed using an acetate buffer (pH 5.22) as the incubation medium. Figure 5A depicts images of the evolution of dry hydrogels during the swelling procedure. Figure 5B includes the data expressed as the percentage swelling degree (calculated from Equation (2)) as a function of time, comparing four formulations.

#### 3.1.6. Field Emission Scanning Electron Microscopy (FESEM) and Energy-Dispersive X-Ray Spectroscopy (EDS)

**Figure 6:**
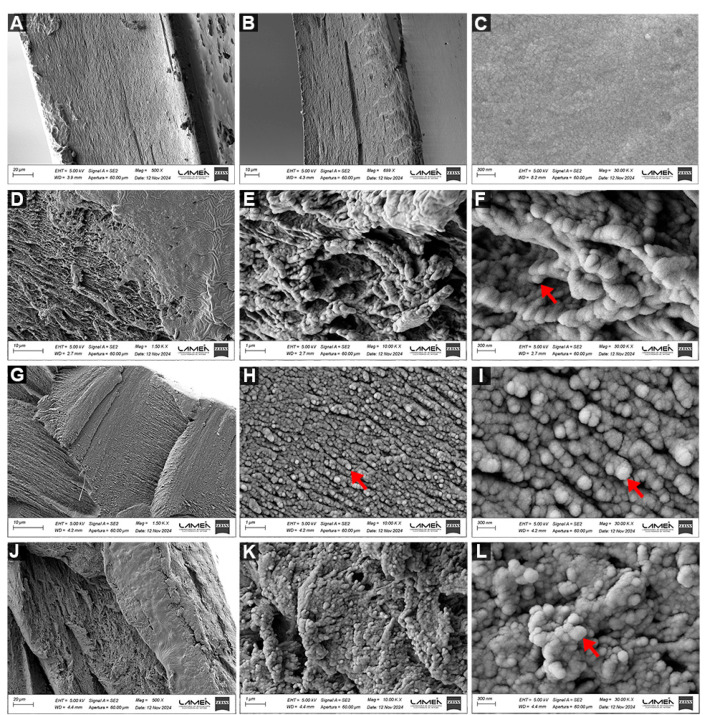
FESEM micrographs, at magnifications of 1500, 10,000 and 30,000×, of the cross-section of H (**A**,**B**) and the surface of H (**C**) and the cross-section of HZ 2.5 (**D**–**F**), HZ 5 (**G**–**I**) and HZ 7.5 (**J**–**L**).

Figure 6 shows the FESEM micrographs of different hydrogels. Figure 6A,B show the cross-section of H, where overlapping layers of the biopolymers forming the matrix can be observed. This sample has a thickness of 144.1 µm. Figure 6C shows the surface of the same H sample. Figure 6D–F correspond to HZ 2.5, while Figure 6G–I belong to HZ 5 and Figure 6J–L belong to HZ 7.5. Figure 6G–I depict the images from HZ 5. The figures in Supplementary Material (Figure S2A–C) correspond to the surface view of HZ 7.5.

The EDS analysis of NPs containing hydrogels confirmed the Zn presence (see Figure S2D–F of the Supplementary Materials).

#### 3.1.7. Thermal Properties: Differential Scanning Calorimetry (DSC)

**Figure 7:**
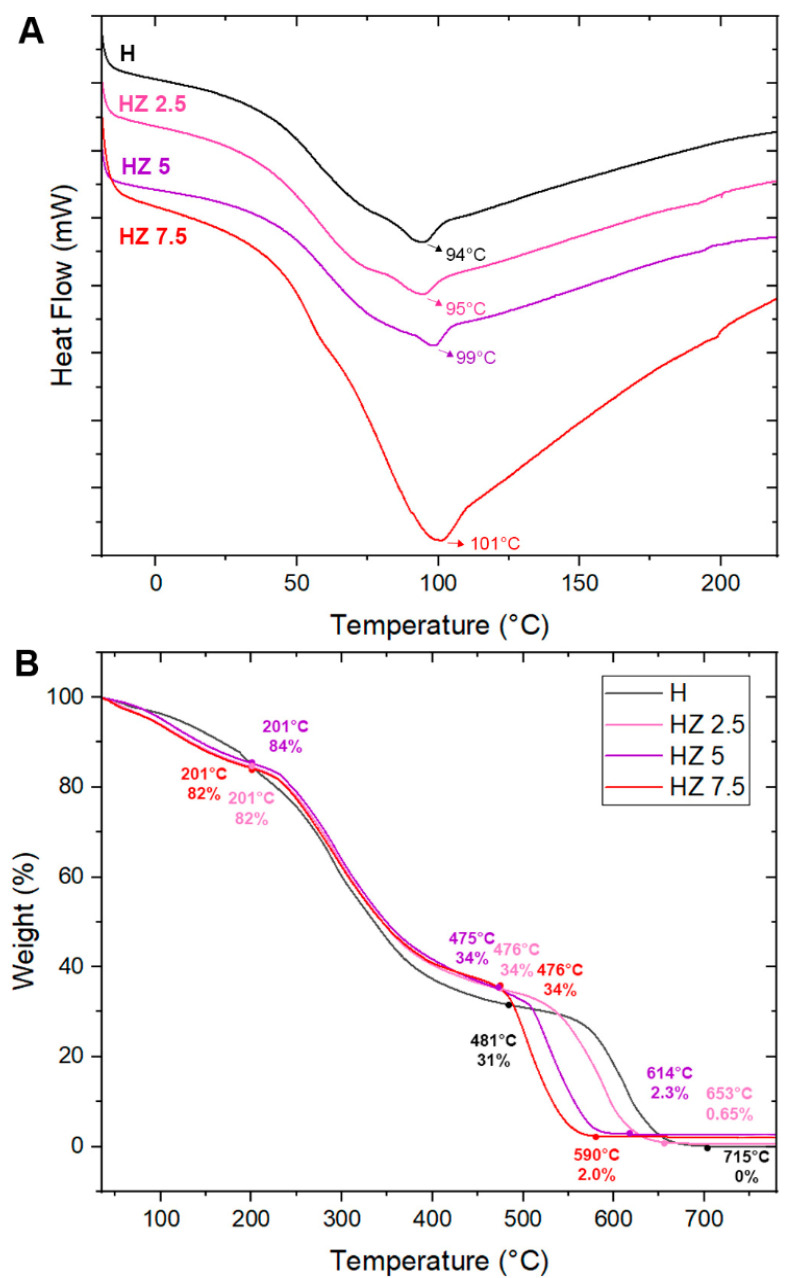
(**A**) DSC of H, HZ 2.5, HZ 5 and HZ 7.5. (**B**) TGA of H, HZ 2.5, HZ 5 and HZ 7.5.

Figure 7A displays the obtained thermograms arising from the analysis of all H samples. The endothermic process is indicated by the downward-pointing arrow, which corresponds to decreasing values along the Y-axis. This direction confirms that all downward peaks are endothermic events in which the sample absorbs heat.

Thermal Properties: Thermogravimetric Analysis (TGA)

As shown in Figure 7A,B, the thermal stability of the samples was analyzed using thermogravimetric analysis (TGA). Table 3 presents the values obtained from TGA analyses of H, HZ 2.5, HZ 5 and HZ 7.5. The DTG curves are included in Figure S3 of the Supplementary Materials.

#### 3.1.8. Flame Atomic Absorption Spectroscopy (FAAS)

Table 4 depicts the data achieved by determining the Zn concentration in each HZ formulation. The data were expressed as % of ZnO (*w*/*w*) and compared with the nominal ZnO proportion and with those estimated through TGA.

### 3.2. Cytotoxity Analysis

Ensuring biosecurity is crucial when creating novel formulations that come into direct contact with the skin. One of the most popular tests in biomedicine for evaluating cytotoxicity is the NRU, which provides quantitative estimates of the number of live cells in a culture. It is predicated on the capacity of living cells to bind and incorporate the neutral red supravital dye into lysosomes. Consequently, the dye cannot be incorporated by non-viable cells [49]. Cell viability was significantly reduced (*** *p* < 0.001) when Vero cells were treated with 1% H_2_O_2_ (positive control for cell death), according to the NRU analysis. Rather, the test showed that there was no difference between the absorbance recorded in the control condition and the cells subjected to various doses (25 to 100%) of the HZ 7.5 hydrogels extracts. It is worth mentioning that this HZ was selected as representative of the rest of the formulations since it contains the highest nominal ZnO NP doses, which are the components of the HZ that could exert some kind of toxicity. The biopolymeric matrix has been explored in other works as biosafe [16].

**Figure 8:**
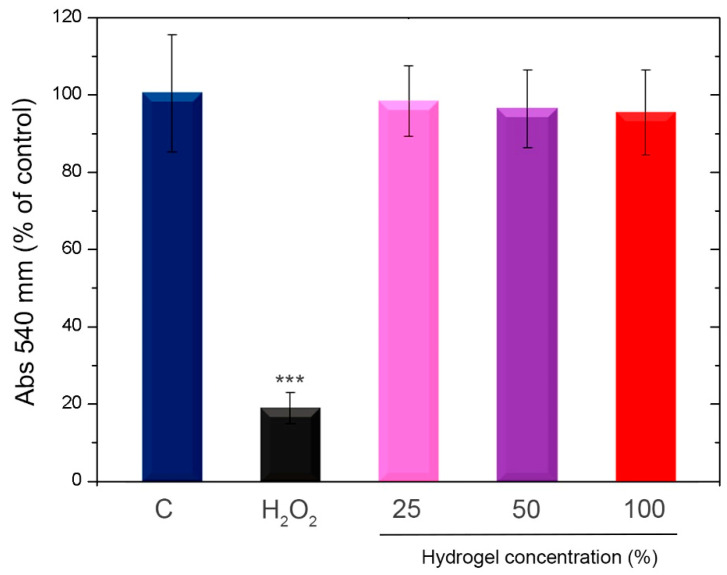
Evaluation of cytotoxicity in vitro. Vero cells were subjected to either 1% hydrogen peroxide (H_2_O_2_) or increasing quantities of the HZ7.5 extracted media for a whole day. The NRU assay was used to assess cell viability. The findings are shown as a percentage in relation to the control of two separate, duplicate tests. C: control condition. SD and means are displayed. *** *p* < 0.001 in comparison to the control.

The achieved findings, included in Figure 8, reveal that hydrogels in all of the evaluated doses do induce a cytotoxic effect on Vero cell cultures.

### 3.3. Virucidal Activity

**Figure 9:**
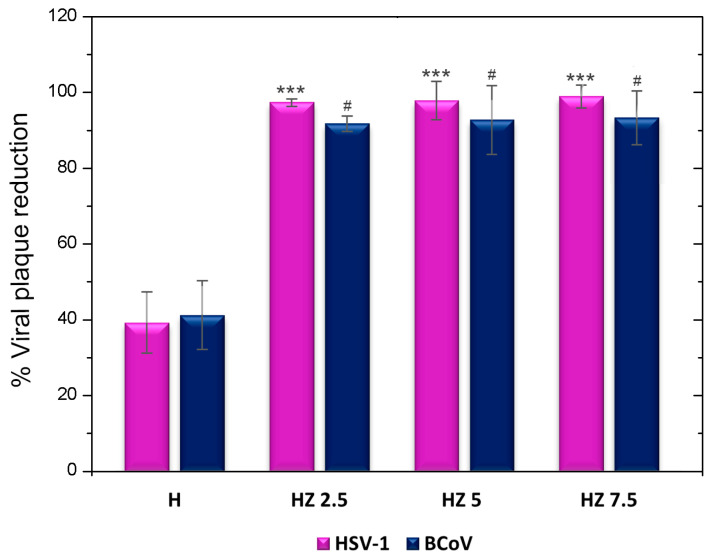
Viral plaque reduction in H, HZ 2.5, HZ 5 and HZ 7.5. *** *p* < 0.001, with respect to HSV-1. # *p* < 0.05, with respect to BCoV.

The study of antiviral activity through virucidal assays was ruled by the potential of these hydrogels for topical applications. In this type of study, the activity of the hydrogels on the viruses before exposing them to contact with cells is evaluated. The virucidal activity of H and the three formulations with different concentrations of ZnO NPs were evaluated using BCoV and HSV-1. The achieved data are included in Figure 9.

### 3.4. Antioxidant Activity

**Figure 10:**
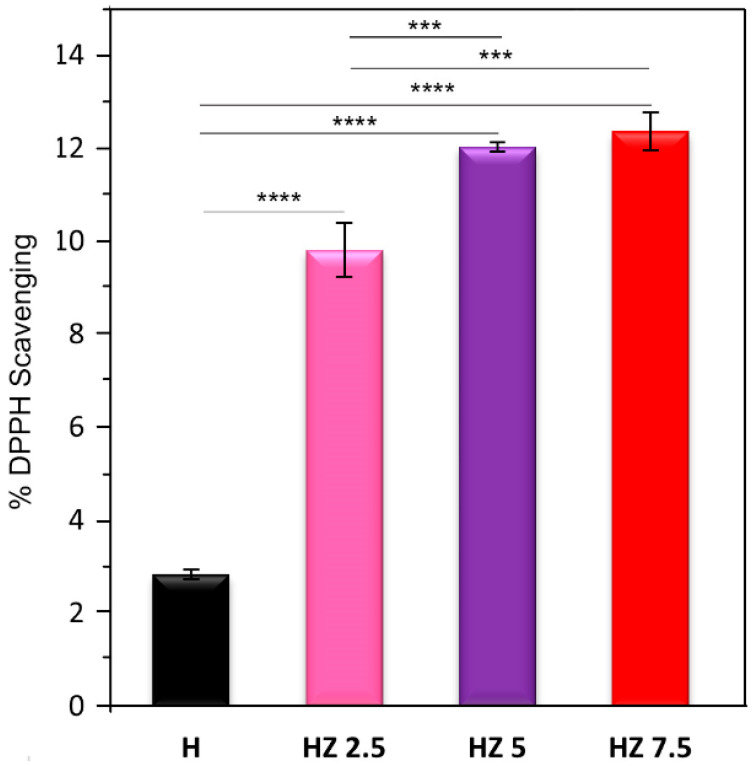
% DPPH Scavenging of H, HZ 2.5, HZ 5 and HZ 7.5. *** *p* < 0.001, **** *p* < 0.0001.

The antioxidant activity of the polymeric matrix and hydrogels loaded with ZnO NPs was evaluated using the DPPH free radical scavenging method. Ascorbic acid, used as a qualitative positive control, exhibited an antioxidant activity of 97%. The results obtained are presented in Figure 10.

## 4. Discussion

This work develops a multifunctional hydrogel for managing viral skin disorders by combining G, Ch, AG and ZnO NPs. Since these infections induce oxidative stress and inflammation, the biomaterials designed within this work provide simultaneous antiviral and antioxidant activity, offering an innovative therapeutic approach. Our contribution is novel in systematically exploring how different concentrations of ZnO NPs loaded in H affect key hydrogel properties, including antiviral and antioxidant ones.

The FTIR spectroscopic analysis (Figure 2) shows two characteristic bands at 432 cm^−1^ and 503 cm^−1^ in the spectrum of Z, which could be associated with the Zn–O bond [41,50]. The spectrum of the H matrix shows the typical absorption signals of the biopolymers comprising it. Ch exhibits a broad band at 3300–3500 cm^−1^ corresponding to the stretching of –OH and –NH groups in its structure, and the bands at 1617 and 1560 cm^−1^ can be attributed to amide I and amide II functional groups (C–O stretching coupled with N–H bending modes). A shoulder at 1150 cm^−1^ corresponds to the β(1–4) glycosidic bond of the polysaccharide unit, and a peak at 1080 cm^−1^ is related to C–O–C stretching within the glucose ring [51]. G displays bands near 1623–1274 cm^−1^, ascribed to the vibration mode of amide I, II and III groups. These are compatible with the protein structure of this biopolymer. AG exhibits bands around 3400 cm^−1^ due to –OH stretching and at 1597 cm^−1^ and 1406 cm^−1^, associated with asymmetric and symmetric carboxylate stretching, respectively. The signal located at 1016 cm^−1^ can be assigned as well to the C–O–C stretching band [52], whereas the band at 2900 cm^−1^ is attributed to the asymmetric stretching vibration of the –CH groups [50]. The incorporation of different concentrations of ZnO NPs in the H matrix did not cause significant differences in terms of the signals appearing in the FTIR spectra. A slight shift toward lower wavenumbers is observed in the band corresponding to the N–H and O–H groups as the concentration of ZnO NPs increases. This means that, in the H spectrum, this band is centered at 3483 cm^−1^ and shifts to 3473 cm^−1^, 3412 cm^−1^ and 3315 cm^−1^ for HZ 2.5, HZ 5 and HZ 7.5, respectively. This behavior could be attributed to interactions between the O–H and N–H groups of the polymers and the ZnO NPs, involving the formation of hydrogen bonding and coordination interactions. This behavior is accentuated with increasing ZnO NP content in the matrix. These results are consistent with previous studies [53,54,55,56,57,58]. It is worth mentioning that, even when the nominal concentration of ZnO increases, the signal ascribed to Zn-O bond is almost undetectable. This finding could be explained in terms of the methodology of ZnO NP incorporation in the H matrix. Since the NPs are encapsulated in the H matrix, it is reasonable that strong interactions between the inorganic moieties and polymeric phase take place, altering or masking the characteristic bands of the ZnO nanoparticles [58,59].

In the context of biomedical and pharmaceutical applications, especially considering topical administration, it is relevant to achieve a compromise between the mechanical strength and structural stability of the hydrogel and the rate of biodegradation in the physiological environment. This is fundamental for the design of controlled release systems and to ensure the hydrogel’s functionality over the required duration of its therapeutic application [60]. It can be observed that almost any difference is found between the values of gel fractions arising from the H matrix and those corresponding to HZ 2.5 and HZ 5 (Table 2). However, at the highest concentration of ZnO NPs explored (HZ 7.5), a significant decrease in the gel fraction value (37.1%) is observed. These findings are consistent with those reported by Raafat et al. [61], who developed xanthan gum and polyvinyl alcohol (PVA) hydrogels loaded with different concentrations of ZnO NPs. Their study showed almost the same dependence between NP concentrations and gel fractions, indicating a lower crosslinking density promoted by the NPs. A possible justification of this behavior can be provided considering the NPs’ aggregation trend, which can interfere in the crosslinking process [33].

The polymeric matrix (H) exhibited the lowest solubility time regarding the loaded H, reflecting its weak structure, as was extensively reported [62] (Table 2). It is worth mentioning that the conditions selected for the assay tried to reproduce the cutaneous environment. The incorporation of ZnO NPs significantly prolonged the dissolution time, reinforcing the H microstructure and improving its stability. Some authors have suggested that NPs can locate in the polymer network spaces, reducing its porosity and creating a more compact microstructure, restricting solvent penetration [63]. In addition, it is worth noting that this effect is dose-dependent since the highest explored concentration (7.5%) shows a lower resistance to the dissolution. This behavior can be attributed to the ZnO NPs’ aggregation when using elevated concentrations, generating a less dense and heterogeneous network that facilitates solvent interaction [33].

In this sense, the markedly lower gel fraction (37.1%) and shorter solubility time (48 h) observed for the HZ 7.5 formulation indicate a poor structural integrity of the hydrogel network, which can be attributed to nanoparticle agglomeration interfering with effective polymer crosslinking. From a therapeutic perspective, this reduced stability is expected to limit the residence time of the formulation on the skin, compromising its ability to provide a sustained delivery of bioactive species. This aspect is particularly relevant for topical antiviral therapies, where prolonged contact and controlled release are required to maintain effective concentrations over the course of viral replication or reactivation events, such as those associated with HSV-1 infections. In contrast, HZ 2.5 and HZ 5 exhibited higher gel fractions and an extended stability under skin-mimicking conditions (up to 96 h), suggesting a more robust network capable of supporting sustained therapeutic action. Therefore, while high ZnO NP loadings may enhance certain bioactivities, excessive nanoparticle incorporation negatively impacts structural durability, highlighting the importance of optimizing ZnO NP concentrations to balance bioactivity with the stability requirements of long-term topical antiviral formulations.

In the case of the dry H, the mean contact angle was 93.91°, indicating a low hydrophilicity (Figure 4). It has been reported that G-based films may exhibit high advancing water contact angles, which are associated with a reduced hydrophilicity due to their geometry or interaction with other polymeric moieties [64,65,66]. Similarly, studies on Ch have reported contact angle values indicative of a low hydrophilicity [67,68]. Białopiotrowicz et al. [64] concluded that, during the gel forming process, hydrophilic groups (hydroxyl, carboxyl and amino) tend to orient toward the interior of the film, becoming involved in the formation of the polymeric network, while hydrophobic moieties (aliphatic chains and aryl groups) could remain exposed on the surface. This molecular reorganization can influence the availability to interact with water, leading to an increase in the contact angle [69,70]. Upon the incorporation of ZnO NPs, a slight reduction in the average contact angle was observed, suggesting a slight increase in hydrophilicity relative to the original matrix (Figure 4E). Several authors have attributed this to the ability of ZnO NPs to interact by means of hydrogen bonding with water molecules [71,72]. However, the HZ 7.5 sample exhibited an average contact angle of 103.28°, possibly due to nanoparticle agglomeration on the hydrogel surface, which may hinder the direct interaction of the polymeric matrix with water [33,73]. This result is in line with the data obtained from the above-mentioned characterization techniques (i.e., gel fraction and solubility).

It is important to highlight that, from a topical application perspective, surface wettability plays a critical role in hydrogel spreading, adhesion and retention on the skin. Hydrogels with a moderate hydrophilicity may promote intimate contact with the hydrated stratum corneum through hydrogen bonding and capillary interactions, favoring adhesion and ensuring a prolonged residence time [74,75]. In this regard, the slight reduction in the contact angle observed for HZ 2.5 and HZ 5 suggests an improved interfacial compatibility with skin. Conversely, the higher contact angle measured for HZ 7.5 indicates a reduced surface hydrophilicity, which may hinder effective spreading and skin adherence, potentially limiting its practical applicability as a topical antiviral formulation.

In general, the swelling profiles of the hydrogels are characterized by a rapid initial expansion followed by a plateau, indicating an equilibrium with the buffer medium (Figure 5). Notably, the HZ 2.5 formulation exhibited the highest swelling percentage, even over H. A trend in the swelling reduction when ZnO NPs increased was observed. A similar tendency has been reported in the open literature. Li et al. [33] developed hydrogels based on urea, acrylamide and choline chloride containing different concentrations of ZnO NPs, revealing that the NP incorporation initially expanded the hydrogel network, enhancing water molecule adsorption. However, as the ZnO NP content increased from 0.4% *w*/*w* to 1.2% *w*/*w*, the swelling capacity significantly declined. The work of Can et al. [34], devoted to the preparation of PVA/chitosan hydrogels, supports these findings. They found that low ZnO NP concentrations improved swelling due to increased network porosity, facilitating water uptake. In contrast, higher nanoparticle doses led to a reduction in swelling degree, likely due to nanoparticle aggregation, which blocked pores, decreasing the availability of hydrophilic sites for water uptake. In the frame of intended applications of these HGs, maintaining a moist environment facilitates the penetration of active ingredients and prevents microbial infections, among other advantages [76]. The ability to control the degree of swelling through ZnO NP doses appears essential for designing hydrogels with tailored dermal properties. An optimal concentration can improve exudate absorption, while an excessive concentration may compromise the hydrogel’s functionality. For instance, in controlled drug delivery systems, reduced swelling can slow drug diffusion, allowing for a sustained release [77]. The figures included in the Supplementary Material (Figure S2A–C), corresponding to the surface view of HZ 7.5, reinforce the existence of NP agglomeration [78].

In the FESEM micrographs of the hydrogel with the lowest nanoparticle concentration (HZ 2.5), shown in Figure 6D–F, a polymeric network with irregular pores is observed when analyzing the cross-section. Evidence of significant NP agglomeration is not detected. Those taken from the cross-section of HZ 5 (Figure 6G–I) exhibit an almost homogeneous distribution of ZnO NPs with a lower porous density when compared with HZ 2.5 images. Figure 6J–L show the HZ 7.5 morphology, revealing a heterogeneous structure where the aggregation of ZnO NPs is clearly detected. To better illustrate the aggregation level, extra SEM images of HZ7.5 are further included in Figure S2A–C. These data are consistent with those arising from other characterization techniques and with some published research [60]. The EDS analysis of different HZs demonstrated the uniform distribution of ZnO NPs, which is more evident with increasing NP proportions (see Figure S2D–F). Although the present FESEM analysis is qualitative in nature, the observed microstructural trends are consistent with the results obtained from independent characterization techniques, including the swelling degree, gel fraction, solubility, wettability and FAAS measurements. These combined results support a coherent structure–property relationship and are in agreement with previous reports describing nanoparticle-induced network densification and aggregation phenomena in polymeric hydrogels [60]. Consequently, FESEM offers qualitative insight into microstructural changes supported by macroscopic, structural and physicochemical results.

Upon the analysis of the heating scan, the association of the biopolymers exhibits maximum heat absorption between approximately 101 and 110 °C. These endothermic peaks are associated with the evaporation of free water bound to hydrophilic groups within the H [79,80]. As shown in Figure 7A, an increase in Tg is observed with rising ZnO NP concentrations in the H matrix, reaching values of 94 °C, 95 °C, 99 °C and 101 °C for H, HZ 2.5, HZ 5 and HZ 7.5, respectively. This behavior is likely due to a combination of factors, including restricted polymer chain mobility, polymer–nanoparticle interactions and the formation of a more rigid structure within the composite system [73]. Moreover, the endothermic peak of the HZ 7.5 sample is characterized by a greater height and width, which is associated with the higher ZnO NP content. This increase in transition enthalpy likely results from a more restricted polymer chain mobility and improved thermal stabilization due to the presence of ZnO [73].

The thermogravimetric curves (Figure 7B) exhibit a similar mass loss trend, with more than one decomposition stage for all four samples. The thermogram of the H matrix displays three distinct weight loss steps, consistent with previous reports where Ch, G and AG polymers were analyzed [81,82]. In the first stage, a loss of 20% of the H mass is observed at 205 °C. This stage is attributed to the volatilization of small molecules, residual acetic acid from the hydrogel synthesis and dehydration of the polymers (25–250 °C) [83,84]. The amount of water absorbed by the polymers is closely related to the availability of amino and hydroxyl groups that form hydrogen bonds with water molecules [85]. The second decomposition stage ends at 481 °C, corresponding to a 69% weight loss. This stage includes the decomposition/depolymerization of Ch chains through the deacetylation and cleavage of glycosidic bonds, initially affecting 2-amino-2-deoxy-D-glucopyranose (Glc) units (130–310 °C), followed by the decomposition of 2-acetamido-2-deoxy-D-glucopyranose (GlcNAc) units (310–400 °C) [85,86,87]. Additionally, the degradation of G peptide bonds (250–350 °C) [84] and polysaccharides in AG (300–480 °C) [88] occurs in this step. The second stage corresponds to the thermal degradation of the pyranose ring in Ch and residual organic materials [81,82,85,86,89], ending at 715 °C with complete mass loss, because this sample is made up entirely of polymers that degrade in the studied temperature range. The thermograms corresponding to HZ 2.5, HZ 5 and HZ 7.5 show three stages of weight loss. In all three, the initial stage starts at 35 °C and ends at 201 °C, with a mass loss of 18%, 16% and 19% for HZ 2.5, HZ 5 and HZ 7.5, respectively, attributed to water trapped within the hydrogel network [85]. The second stage shows mass losses of 66.0%, 65.8% and 66% at 476, 475 and 476 °C for the same samples, respectively. These samples exhibit a slightly lower mass loss during this stage, when comparing them with the hydrogel without ZnO NPs. This is likely due to the interactions between ZnO and the hydroxyl groups of the biopolymers, which delay polymer degradation. In the presence of ZnO NPs, the final decomposition step ends with residual masses of 0.65%, 2.3% and 2.0% for HZ 2.5, HZ 5 and HZ 7.5, respectively. It is worth mentioning that these data could be considered to estimate the HZ composition in terms of ZnO content. From this perspective, it is assumed that, as an estimation, a complementary quantitative technique is always required to assess the reliable and accurate determination of ZnO in the HZ matrix. Therefore, the data in Table 4, where the results are compared with those achieved using FAAS, demonstrate a good concordance with the nominal proportions of ZnO NPs added during the synthesis, which reveals a high efficiency in the loading procedure. In the case of TGA, relatively coherent results are observed in the case of HZ 2.5 and HZ 5, in terms of both nominal and FAAS data. In the case of HZ 5, the significant difference observed could be attributed to operative mistakes relative to the measurements in the TG equipment.

The DTG curves (Figure S3) confirm that sample decomposition occurs in three distinct stages. This suggests that, above 480 °C, the presence of ZnO NPs adversely affects the thermal stability of the hydrogel matrix. These findings are consistent with those reported by Lizundia et al. [90], who attribute this behavior to a catalytic effect of ZnO NPs on the thermal degradation of the polymer. However, considering that the hydrogels are intended for topical application to treat skin infections, this thermal behavior is not relevant under physiological temperature conditions.

The cytotoxicity of hydrogels in Vero cells was studied and is shown in Figure 8. Studies by other authors [91,92], including our own [93], have shown that Ch is a non-cytotoxic compound. On the other hand, the low cytotoxicity of ZnO NPs has also been demonstrated by other authors, proposing its use in multiple biomedical applications [94,95,96]. Our results demonstrated that cell viability was not affected, according to the NRU assays performed with our hydrogels. In addition, our own previous work involving ZnO NPs has demonstrated their biosafety using an almost similar assay [97].

The antiviral activity of the biopolymers composing the H matrix has been earlier reported. Ch has demonstrated antiviral properties through its cationic charges interacting with enveloped viruses, thereby altering their permeability and blocking viral entry into host cells. Its antiviral activity is believed to stem from direct interactions with viral particles, such as the spike (S) protein in coronaviruses [98]. The synergy of Ch with inorganic nanoparticles (Cu and Ag) has been demonstrated, as revealed in our own previous work [16,44]. In the case of G, some recent articles indicate antiviral activity when this biopolymer is linked to inorganic nanoparticles [99,100]. The incorporation of ZnO NPs into the matrix potentiates the viral activity reduction capacity of the H-based formulations (Figure 9). The antiviral properties of ZnO have been reported for various viruses, including rhinoviruses, human immunodeficiency virus (HIV), equine arteritis virus (EAV), dengue virus (DENV), hepatitis E (HEV) and C (HCV) viruses, influenza A virus (IAV), respiratory syncytial virus (RSV), herpes simplex viruses (HSV-1 and HSV-2) and severe acute respiratory syndrome coronavirus 2 (SARS-CoV-2) [30,99,101,102,103,104]. Previous work has shown that zinc oxides can trap viral particles, preventing them from infecting cells. Other authors argue that the antiviral action of ZnO is mainly through the inhibition of viral replication. However, given the design of our experiments, it is possible to suggest that, in HZ hydrogels, ZnO significantly enhances the virucidal effect exerted by the raw H by attaching to and trapping the virus, breaking its entry into the host cell and inhibiting the viral infection. While the hydrogels were effective against HSV-1, a virus closely linked to skin diseases, it was shown that their action could also be effective against other viruses, such as coronavirus. These results are interesting given that it is known that SARS-CoV 2 can spread through fomites, and its short- and long-term consequences in humans are still being studied; for example, it is currently known that this virus can directly infect keratinocytes, causing some skin disorders

The polymeric matrix H showed a low antioxidant activity against the DPPH radical (Figure 10), with a scavenging rate of 2.83%. Ch is primarily responsible for the antioxidant effect of H, due to its amino and hydroxyl groups, which can act as reducing agents by donating a hydrogen atom or an electron to DPPH [13,105,106,107]. It has been reported that G and AG exhibit low intrinsic antioxidant activity, which can only be enhanced through hydrolysis or the incorporation of phenolic compounds [108,109,110]. This limited DPPH scavenging capacity may also be attributed to the reduced availability of active functional groups following polymer crosslinking [109]. The samples loaded with different nanoparticle doses exhibit an enhanced antioxidant capacity compared to the unloaded matrix. The ANOVA analysis showed highly significant differences (*p* < 0.0001) in the antioxidant activity between H and ZnO NP-loaded formulations, except between HZ 5 and HZ 7.5, where no significant differences were observed. This improvement is attributed to ZnO NPs, which can facilitate electron density transfer from the oxygen atoms to the unpaired electron on the nitrogen atom of the DPPH molecule, thereby reducing the intensity of the n→π* transition at 517 nm [110]. It is worth noting that the DPPH assay primarily reflects the ability of a material to act as an electron or hydrogen donor and is commonly employed as an initial screening method for comparing antioxidant performance among related systems under identical conditions.

Previous studies have demonstrated that the DPPH scavenging activity of ZnO NPs increases in a dose-dependent manner [111,112]. Zafar et al. [113] developed hydrogels composed of carboxymethylcellulose (CMC) and G loaded with ZnO NPs for sustainable food packaging applications. Through the DPPH scavenging assay, they observed that CMC/G/ZnO nanocomposites exhibited a greater antioxidant activity than the individual polymers, and that free radical scavenging capacity was dose-dependent. The reduction in reactive oxygen species (ROS) is particularly relevant for topical applications, as the accumulation of free radicals during inflammatory or infectious skin processes contributes to tissue damage. Therefore, even a moderate antioxidant response, as evidenced by the DPPH assay, may contribute to protecting the polymeric network and mitigating local oxidative stress, thereby supporting tissue recovery [114].

## 5. Conclusions

Multifunctional hydrogels based on G, Ch and AG loaded with ZnO NPs were successfully prepared through simple, low-cost and eco-friendly methods. The incorporation of ZnO NPs induced concentration-dependent changes in the structural, surface, thermal, antiviral and antioxidant properties of the hydrogels. The lowest explored doses of ZnO NPs (HZ 2.5) induced the highest swelling degree (1253%), over the level reached for the matrix (1044%). As the ZnO NP concentration increased, a progressive reduction in swelling capacity was observed, accompanied by pore blockage and increasing morphological heterogeneity, as confirmed by FESEM. The highest explored doses of NPs (HZ 7.5) promoted a marked decrease in gel fraction (37.1%) compared to the H matrix (59.5%), indicating a reduction in the effective crosslinking density associated with nanoparticle aggregation. Surface wettability measurements supported these trends, showing a decrease in the contact angle at low ZnO NP concentrations (84.23° for HZ 2.5 and 91.88° for HZ 5) relative to the unloaded matrix (93.91°) and an increase for HZ 7.5 (103.28°), reflecting a reduction in surface hydrophilicity. The FTIR analysis confirmed the interactions between ZnO NPs and the functional groups of the polymeric matrix, while EDS mapping verified their effective distribution within the hydrogels. Thermal analyses revealed an increased network rigidity upon ZnO NP incorporation, evidenced by a progressive rise in glass transition temperature. Consistently, TGA/DTG results showed the presence of inorganic residues corresponding to ZnO NPs and a slight delay in polymer degradation at intermediate nanoparticle concentrations. Moreover, ZnO NP incorporation extended the hydrogel’s degradation time under skin-mimicking conditions (32 °C, pH 5.22), although excessive nanoparticle loading compromised network resistance. All ZnO-loaded hydrogels exhibited improved virucidal activity against herpes simplex virus type 1 (HSV-1) and bovine coronavirus (BCoV), demonstrating their potential for topical antiviral applications. A significant improvement in terms of virucidal efficacy was evidenced when compared to the data from HZ with the corresponding bare H. Finally, the presence of ZnO NPs markedly enhanced the antioxidant capacity of the hydrogels, with statistically significant improvements compared to the unloaded matrix (*p* < 0.0001).

Overall, these findings suggest the existence of an optimal ZnO NP concentration range (between 2.5 and 5% *w*/*w*) that balances enhanced stability and antiviral potential while preserving structural and swelling properties essential for effective application in skin treatments for viral infections.

## Figures and Tables

**Table 1:** Nominal composition, expressed as %*w*/*w*, of different hydrogel formulations.

Sample	Ch (% *w*/*w*)	G (% *w*/*w*)	AG (% *w*/*w*)	ZnO NPs Relative to Ch (% *w*/*w*)	ZnO NPs (% *w*/*w*)
**H**	30.30	60.60	9.10	-	-
**HZ 2.5**	30.07	60.15	9.02	2.5	0.75
**HZ 5**	29.85	59.70	8.95	5	1.50
**HZ 7.5**	29.63	59.26	8.88	7.5	2.23

**Table 2:** Gel fraction (%) and solubility time (hours) of H, HZ 2.5, HZ 5 and HZ 7.5.

Sample	GF (%)	Solubility (h)
**H**	59.5	14
**HZ 2.5**	62.0	96
**HZ 5**	59.7	96
**HZ 7.5**	37.1	48

**Table 3:** Temperature range and mass loss for H, HZ 2.5, HZ 5 and HZ 7.5 obtained from TGA, and temperatures corresponding to the maximum degradation rate obtained from DTG.

Sample	Degradation Stage	Temperature Range (°C)	Mass Loss (%)	Temperature of Highest Degradation Rate (°C)
H	1°	35–481	69	290
	2°	481–715	100	613
HZ 2.5	1°	35–201	18	113
	2°	201–476	66	292
	3°	476–590	99.35	591
HZ 5	1°	35–201	16	114
	2°	201–475	66	292
	3°	475–614	97.7	527
HZ 7.5	1°	35–201	18	115
	2°	201–476	66	289
	3°	476–590	98	503

**Table 4:** Nominal concentration of ZnO NPs (% *w*/*w*) and data of ZnO NPs obtained using FAAS (% *w*/*w*) and TGA (% *w*/*w*).

HZ	Nominal ZnO NPs (% *w*/*w*)	ZnO by FAAS (% *w*/*w*)	ZnO by TGA (% *w*/*w*)
**HZ2.5**	0.75	0.63	0.65
**HZ5**	1.50	1.40	2.30
**HZ7.5**	2.23	2.06	2.00

## Data Availability

The original contributions presented in this study are included in the article/Supplementary Materials. Further inquiries can be directed to the corresponding author.

## Supplementary Materials

The following supporting information can be downloaded at https://www.mdpi.com/article/10.3390/v18010076/s1. Figure S1. Structural, physicochemical and surface properties of ZnO NPs used within this work. A. XRD patterns of ZnO (ZQ). B. Transmission Electron Microscopy (TEM) micrograph of the ZnO NPs. C. Summary of physicochemical parameters, including band gap energy, crystallite size (nm), hydrodynamic diameter (mm), polydispersity index, zeta potential (mV) and pH of the ZnO NP suspension; Figure S2: Surface of HZ 7.5 (A–C) to show the clusters of ZnO NPs. EDS of HZ 2.5 (D), HZ 5 (E) and HZ 7.5 (F), showing the distribution of ZnO NPs across the H matrix in each HZ; Figure S3: DTG of H, HZ 2.5, HZ 5 and HZ 7.5.
