# Supplementary material for: Polymeric Hydrogels Loaded with ZnO Nanoparticles as Promising Tools for Tacking Viral Skin Disorders
Source: Viruses. 2026 Jan 5;18(1):76. doi: 10.3390/v18010076 (PMC12846630; doi:10.3390/v18010076)
Supplement: Supplementary file 1 [file viruses-18-00076-s001.zip › viruses-4049243-supplementary.pdf]

Supplementary material

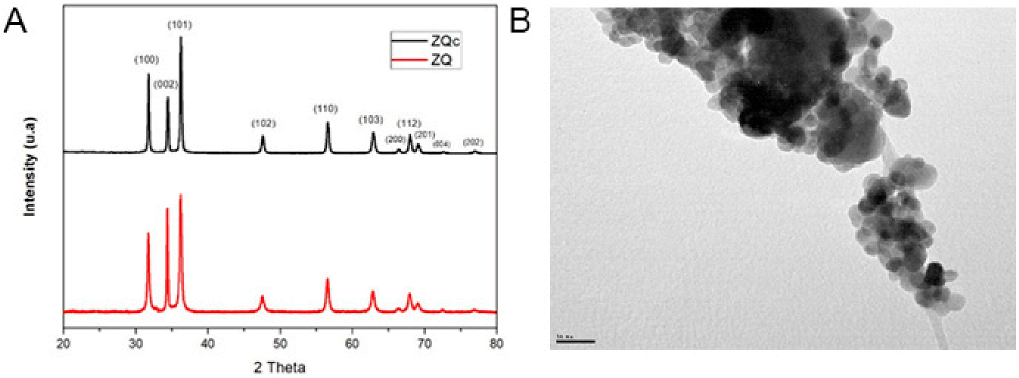

C

NPs	Band Gap (Eg)*	Crystallite size (nm)	Hydrodinamic diameter (mm)	Polydispersion index	Zeta potential (mV)	pH
ZnO NPs	3.06 eV	46.6	707.8 ± 30.0	0.575	-13.5 ± 0.2	9.03

*0,5 mg /10 mL distilled water

Figure S1. Structural, physicochemical and surface properties of ZnO NPs used within this work. A. XRD patterns of ZnO (ZQ). B. Transmission Electron Microscopy (TEM) micrograph of the ZnO NPs. C. Summary of physicochemical parameters, including band gap energy, crystallite size (nm), hydrodynamic diameter (mm), polydispersity index, zeta potential (mV), and pH of the ZnO NPs suspension.

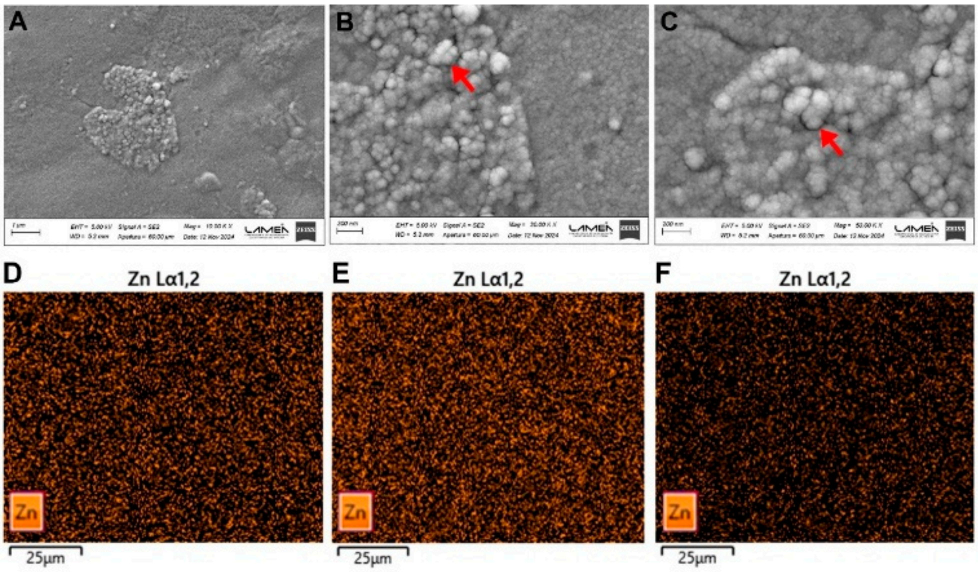

Figure S2. Surface of HZ 7.5 (A-C) to show the clusters of ZnO NPs. EDS of HZ 2.5 (D), HZ 5 (E) and HZ 7.5 (F) showing the distribution of ZnO NPs across the H matrix in each HZ.

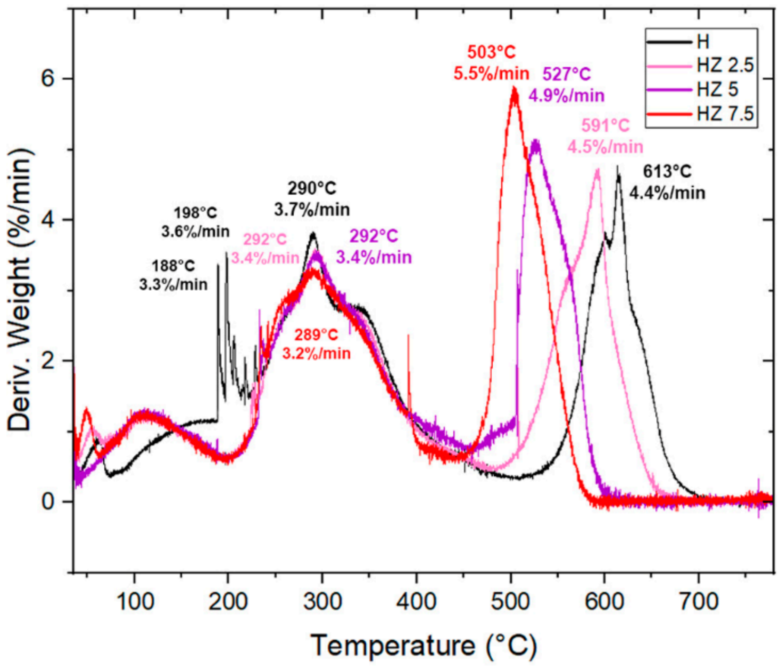

Figure S3. DTG of H, HZ 2.5, HZ 5 and HZ 7.5.
